# Supplementary material for: Occupational burnout during war: The role of stress, disruptions in routine, sleep, work-family conflict, and organizational support as a moderator
Source: PLoS One. 2025 Jan 24;20(1):e0316917. doi: 10.1371/journal.pone.0316917 (PMC11759394; doi:10.1371/journal.pone.0316917)
Supplement: Attachment — Submitted filename: Response to Reviewers.docx [file pone.0316917.s001.docx]

*Occupational burnout during war: The role of disruptions in routine, sleep, work-family conflict, and organizational support as a moderator*

**Response to Reviewers**

Reviewer #1

| Line 37: the main objective of the current research was to address the literature gap regarding burnout during wartime, which is not present clearly in the research paper. | Thank you for your comment. In the introduction, we have added a note that most literature on wartime situations focuses on emotional responses and the emergence of mental health issues, often giving less consideration to the psychological reactions from a unique occupational perspective (lines 19-22). Additionally, in the discussion section, we included a reference emphasizing that work can serve as a crucial space for preventing long-term psychological and functional damage. |
| --- | --- |
| A major concern is the clarity regarding participant selection, such as how they were recruited and the specific inclusion and exclusion criteria applied. | The inclusion and exclusion criteria were individuals aged 28-60 who are employed full-time or part-time, have not been in military service, and have not been evacuated from their homes due to the war. This information has been added in line 209 in the participants' section.  The participants were recruited by the researchers and research assistants through snowball sampling via WhatsApp groups, dedicated Facebook groups, and similar platforms, as mentioned in lines 225-226.  . |
| Line 205: Re-check with data in Table 1. It is suggested to address One missing participant. | Apologies for the typo, it has now been corrected. We had 261 women, 112 men and one participant of unknown gender. |
| Line 207: It is suggested to specify the type of employees involved in this research, including their job functions and tasks, as different jobs and tasks result in different levels of stress. | We do not have information regarding the participants' specific roles, as our focus was on examining burnout and stress levels in the context of war without concentrating on any particular sector. During the war, the entire economy shifted into emergency mode, with all sectors operating under extreme pressure. Consequently, traditional sectoral differences in stress and burnout became less relevant in the context of acute wartime conditions. However, we believe that longitudinal studies conducted during wartime should consider sectoral differences. We have noted this in the study’s limitations and included it as a recommendation for future research (lines 483). |
| Line 209: It is suggested to specify the timeframe, such as having sustained a physical injury within 2 months or within 1 year and specify the severity of the physical injuries being counted. | These questions relate to injuries incurred during the war that began on October 7th, with data collected one month later. We added information that the questions focused on injuries occurring during the war period (line 213). |
| In Table 1 - Gender : It is suggested to re-check one Adding participant (Line 205). | We have added the information regarding the participant with unknown gender. |
| Age : Is there any information on the average age, minimum age, and maximum age of the participants? This is an unequal in each age group; 28-34 (=8), 35-40(=5) | The participants reported their age by selecting the age group to which they belong. We chose this method to avoid missing data or incorrect information. All age groups were in 5-year increments, except for the first group, which was extended down to age 28. We thought it would be beneficial to expand this, as this age range in Israel is common for individuals seeking their first stable employment. The overall range was 28 to 60. |
| Higher Education : It should be categorized by educational level: bachelor's degree, master's degree, and doctorate degree. | We have added to Table 1 the levels of higher education as suggested. |
| Organization Variable : This part should be clarified further: What is the Organization Variable? Are these related to Job Characteristics? | The organizational variables refer to the different types of support the organization provided during the war. You can find the details in line 238. We included this information here to avoid adding another table to the document. |
| Line 222 : What is the inclusion/exclusion criteria for this study ? | The inclusion and exclusion criteria were individuals aged 28-60 who are employed full-time or part-time, have not been in military service, and have not been evacuated from their homes due to the war. This information has been added in line 209 in the participants' section. |
| Line 235 : Has the nature of this work changed compared to how it was before the war? | Unfortunately, the current study does not include earlier measurements. These questions specifically address how organizations responded to the war. As detailed in lines 240: ' During the war, my organization provided:' |
| Line 259 : What does MIDUS stand for? | This is merely a reference to the reporting on the tool we used in the study. The questionnaire was developed as part of the MIDUS study; we have no affiliation with that research, but we have utilized its tool. |

Reviewer #2:

| Reviewer comments | Authors' revision |
| --- | --- |
| According to Table 4, Predictors of Occupational burnout during war are  (1) stress (2) Work to family conflict (3) Family to work Conflict (4) Sleep disruption (5) Routine disruption. And organizatonal support is the moderator. Thus, the recommended topic is  "Stress, Work to Family Conflict, Family to work Conflict, Sleep disruption and Routine disruption Predicting Occupational Burnout during war: The Moderator of Organizational Support". | Thank you for the suggestion. We have added 'stress' to our title:  'Occupational burnout during war: The role of stress, disruptions in routine, sleep, work-family conflict, and organizational support as a moderator |
| In the abstract participants completed a questionnaire assessing demographic details, and variables should be 1) stress (2) Work to family conflict (3) Family to work Conflict (4) Sleep disruption (5) Routine disruption. And organizational support is the moderator. Please add data analysis in the abstract. | We have added information about the data analysis in the abstract as requested. |
| Please use researchers instead of we or our. | Thank you for your comment. We have revised the abstract and manuscript, replacing "we" and "our" with more general terms like "the researchers". |
| The introduction, literature review, methods, results, discussion, conclusion, limitations, and references are okay. | Thank you. |

Reviewer #3:

| Reviewer comments | Authors' revision |
| --- | --- |
| The manuscript is well-structured, technically competent, and contributes to wartime occupational burnout research. The study rigorously tests its hypotheses using hierarchical regression and moderation analysis. Well-reported data support conclusions. To ensure openness and data-sharing compliance, all underlying data are available. The article uses ordinary English to define fundamental ideas and explain theoretical frameworks. Dual publication, research ethics, and publishing ethics seem fine. Overall, the text offers significant insights for crisis employee burnout management. The study followed ethical guidelines and received institutional review board approval, thus there are no ethical problems. The study offers data ethically and with integrity. Future studies should examine burnout's long-term effects and interventions. | Thank you very much. |
| I advocate publishing this work following minor edits to clarify parts like the practical consequences of the findings. | Thank you for your comment. The discussion section highlights the role of work as a space for healing and recovery during crises. We have added further references in lines 457-463, and there is also a reference in the conclusions (lines 496-506). |
| This study advances occupational burnout theory and practice in war-torn areas. | Thank you. |

Reviewer #4:

| Reviewer comments | Authors' revision |
| --- | --- |
| The manuscript covers an important and novel topic by exploring burnout during war conditions with relevant factors. However, this study still lacks several important factors, such as occupation and daily working hours. | We do not have information regarding the participants' specific roles, as our focus was on examining burnout and stress levels in the context of war without concentrating on any particular sector. A requirement for participation in the study was working part-time to full-time. The key factor was maintaining continuous employment within a specific timeframe during the war, as opposed to those who completely stopped working. During the war, the entire economy shifted into emergency mode, with all sectors operating under extreme pressure. Consequently, traditional sectoral and occupational differences in stress and burnout became less relevant in the context of acute wartime conditions. However, we believe that longitudinal studies conducted during wartime should consider sectoral and occupational differences. We have noted this in the study’s limitations and included it as a recommendation for future research (lines 483-485). |
| Measuring occupational burnout after the occurrence of war may not be appropriate, as it involves post-traumatic stress disorder. Therefore, the findings may not accurately represent employees’ occupational burnout. | Thank you for your comment. In this study, we focused on a population not directly affected by the war, which is why we chose to measure stress as a control variable rather than trauma. Our results show burnout levels beyond stress. Additionally, since the data was collected about a month after the war began, a time typically associated with acute stress symptoms rather than PTSD, we felt this approach was more appropriate. That said, we recognize the importance of addressing PTSD in future research, and we have added this recommendation to the discussion section (line 471). |
| I would like the author to consider expanding the discussion on why focusing on burnout during war is particularly important and how it could be relevant to policymakers, organizations, and family members. | Thank you for your comment. The discussion section highlights the role of work as a space for healing and recovery during crises. We have added further references in lines 460-463, and there is also a reference in the conclusions (lines 496-506). |
