# Supplementary material for: Occupational burnout during war: The role of stress, disruptions in routine, sleep, work-family conflict, and organizational support as a moderator
Source: PLoS One. 2025 Jan 24;20(1):e0316917. doi: 10.1371/journal.pone.0316917 (PMC11759394; doi:10.1371/journal.pone.0316917)
Supplement: Attachment — Submitted filename: Response to Reviewers1.11.docx [file pone.0316917.s002.docx]

Occupational burnout during war: The role of stress, disruptions in routine, sleep, work-family conflict, and organizational support as a moderator

Dear Prof. Siripipatthanakul,

Thank you for your comments.

Please find our responses to each of the points raised in the review.

Best Regards,

Maor & Irene

**Response to the Editor Comments:**

(1) Kindly follow reviewer 1's comments.

**Thank you, we reviewed all of Reviewer 1's comments and responded to each accordingly.**
(2) In the abstract, please add that hierarchical linear regression and Pearson Correlation were utilized to model the outcome variable of organizational burnout.

**Done**.
(3) In The last paragraph of the introduction, please add: Thus, this study identified the predictors of occupational burnout based on stress, disruptions in routine, sleep, work-family conflict, and organizational support as a moderator (Line 48).

**Done.**
(4) Kindly check capital letters, such as occupational Burnout and Process Model 1 (in the abstract); it should be "occupational burnout and Process Model 1", or "Occupational Burnout and Process Model 1".

**Thank you for the comment, it has been corrected.**
(5) Table 2. Please add: The significant correlated factors of occupational burnout are stress (r=.63**), work-to-family conflict (.47**), sleep disruption (r=.44*), family fo work conflict (r=.42**), and routine disruption (r=-.322**), respectively.

**Thank you for your comment. We added a verbal report on the correlations in the Results section, referring to Table 2. The correlation between burnout and routine disruption is r=.35, which we assume is what you were referring to, and we have made the necessary correction.**
(6) Table 3. Step 2: Please add that the most influencing factors of occupational burnout are stress (β=.43**), work to family (β=.15**), routine disruption (β=-.14**), sleep disruption (β=0.98**), and family fo work (β=0.07), at 2= .48 (occupational burnout explanation power is 48%). Kindly check if gender is added, which may reflect different results with Pearson Correlation in Table 2 in important priority.

**Thank you for your comment. We have added a reference to the regression findings in the results section. Additionally, gender was carefully considered, as the relationship between gender and burnout is presented in Table 1, highlighting a significant difference in burnout levels between men and women. As a result, we controlled for gender in the first step of the regression analysis, ensuring a comprehensive understanding of the study's objective of explaining burnout during wartime.**
(7) Table 4. Please use Predictors instead of Predictor.

**Done.**
(8) Discussion and conclusion should summarize the findings, such as,
The significant correlated factors of occupational burnout are stress (r=.63**), work-to-family conflict (.47**), sleep disruption (r=.44*), family fo work conflict (r=.42**), and routine disruption (r=-.322**), respectively.
Comparison with regression: the most influencing factors of occupational burnout are stress (β=.43**), work to family (β=.15**), routine disruption (β=-.14**), sleep disruption (β=0.98**), and family fo work (β=0.07), at 2= .48 (occupational burnout explanation power is 48%).

**Thank you for your comment. We have incorporated the information into the results section and emphasized the regression findings in the discussion section**.
(9) Please ensure that the conclusion is made on how strategic planners could improve organizational burnout based on stress, work-to-family conflict, sleep disruption, family-of-work conflict, and routine disruption. (A summarized table of hypotheses is suggested in the result section, if applicable).

**Thank you for your comment. We have expanded the conclusion section to include strategies for reducing organizational burnout based on the findings of the study (line 513). Additionally, in the introduction, we clearly outlined the hypotheses and referred to them systematically in the results section, and particularly in the discussion. We believe this approach will enhance the clarity of the findings.**
(10) Please ensure that volunteering or organizational support is the moderator. Please use only one term or mention anywhere if these two terms are interchangeable.

**Thank you for your comment. In the study, we explored various types of organizational support and their relationship to burnout (Hypothesis 3). We also examined whether the types of support moderated the relationship between work-to-family conflict and burnout and found that only volunteering opportunities, one type of organizational support, did so. The information appears in both the introduction (lines 199-205) and the discussion (line 439).**
(11) Limitations should be added about the time, such as measuring occupational burnout after the occurrence of war; the findings represent employees' occupational burnout perceptions during the data collection period.
**Done.**

Reviewer #1:

1. Rephrase to reflect a more inclusive and respectful tone e.g. Line 208 "unknon gender" --> unreported

**Done**.
2. Line 212 "a month after the war" --> If the exact timeline is important for the study, specifying the start date of the war or elaborating on how long the war had been going on by the time the study was conducted would provide greater clarity.

**The study was conducted one month after the war began, an important detail given that this period is characterized by heightened stress. The war is still ongoing. We have included a reference in the participants' description indicating that additional information about the war can be found in the Recruitment and Procedure section. Additionally, the introduction provides a description of the characteristics of the war and the experiences of those affected by it (line 44).**
3. Table 1: The term 'No' under Higher Education is unclear. Does it refer to no formal education or a level lower than a bachelor's degree?

**We refer to individuals without academic education, so we replaced the term 'No' with 'High School / Professional Diploma.'**
4. Table 1: The organizational variables should be fully detailed. For example, the term 'Information' remains unclear.

**We updated the term in the table to 'Information about the organization' and added a note below the table indicating that full details regarding the nature of the questions can be found in the 'Questions regarding organizational support' section of the Method.**
5. Line 227 "various social media networks" specifying which networks or types of networks (e.g., Facebook, WhatsApp) could offer better insight into how participants were targeted.

**Done.**
6. Line 313 occupational burnout in the war sample" could be simplified to "occupational burnout among participants" for smoother reading.

**Done.**
7. "The criterion variable" is used in one instance, and "the outcome variable" is used earlier. These terms should be consistent throughout.

**We replaced the term "criterion" with "outcome variable."**
8. Line 321 --> "results are presented in Tables 3" refer to "Table 3" (singular) for consistency

**Done.**
9. The terms "stress," "work-to-family conflict," "routine disruptions," and "sleep disruptions" are inconsistently capitalized.

**Thank you for the comment; we have made the necessary revisions accordingly.**
10. The finding that "stress, work-to-family conflict, routine disruptions, and sleep disruptions" contributed significantly to burnout is important, but the magnitude of these effects (e.g., the beta coefficients or effect sizes) should be briefly mentioned.

**Done.**
11. The explanation of the non-significant findings regarding gender and family-to-work conflict is too brief. Offering a possible interpretation or context for why these variables did not significantly impact burnout could enhance understanding.

**Thank you for the comment. In the Results section, we added information on the strength of the beta coefficients obtained in the regression analysis. In the Discussion, we addressed the lack of contribution from family-work conflict (lines 402–409) to the model as well as the lack of contribution from gender (lines 414–419).**
12. The term "work-to-family conflict" is abbreviated as "WFC" partway through the paragraph, but it is first presented in full. For consistency, either use the abbreviation throughout or introduce the abbreviation earlier.

**Thank you for the comment, we replaced 'WFC' with 'work-to-family conflict,' as it appears throughout the manuscript."**
13. The description of volunteer opportunities as moderating the relationship between WFC and burnout is vague. The phrase "as in higher volunteer opportunities the association...was insignificant" could be clearer. For example, more detail on how this moderation worked would enhance understanding. What exactly about the volunteer opportunities mitigated burnout?

**Thank you for the comment. We added a clarification regarding the significance of the moderation: 'In other words, among employees who perceived their organizations as offering numerous volunteering opportunities during wartime, no significant relationship was found between work-to-family conflict and burnout,' along with a possible explanation for this in lines 454–460.** **...volunteer opportunities during wartime may provide added value, meaning, and a sense of vitality and relevance in the context of the larger situation (war). By doing so, organizational volunteers help reduce the tension individuals feel between their work and family functions, and its association with burnout...**

14. The shift between past and present tense (e.g., "examined" vs. "are presented") feels inconsistent. Keeping the tense consistent throughout the passage would improve flow.

**Thank you for your comment. We have made revisions to ensure that the reporting of the study results is in the past tense.**
15. In the conclusion, outline specific directions for future research, such as how other types of organizational support could be explored or how different crisis settings (e.g., non-war-related crises) might yield different results.
**Thank you for the comment. In addition to addressing future research in the discussion section, we have added a focused reference in the conclusions section (line 523).**

Below are the key suggestions:
1. Clarify Key Terms and Concepts

**Done**
2. Avoid redundancy in the discussion by consolidating similar points. For example, the relationship between routine disruption and burnout is mentioned multiple times. Streamlining this information would enhance readability.

**Thank you for the comment. We revised the discussion section, accordingly, reducing some of the repetitive information about routine disruption and burnout.**

3. Ensure tense consistency (e.g., switching between past and present tense in some sections) to maintain a professional and polished tone.

**Done**
4. Simplify overly complex sentences to enhance clarity. Long, dense sentences can obscure the key message, so aim for concise language where possible.

**Done**
5. Proofread for grammar and syntax errors, ensuring clear communication and a polished final draft.

**Done**

Reviewer #2: Acceptable, however, please note that hypotheses in the literature review should be added because discussion and conclusion could be more precious and comprehensive.

**Thank you for the comment. In the introduction, under the Current research section, the hypotheses are presented. We added numbers to them and, accordingly, referred to them in the discussion based on the numbering to create greater clarity.**
