# Supplementary material for: Occupational burnout during war: The role of stress, disruptions in routine, sleep, work-family conflict, and organizational support as a moderator
Source: PLoS One. 2025 Jan 24;20(1):e0316917. doi: 10.1371/journal.pone.0316917 (PMC11759394; doi:10.1371/journal.pone.0316917)
Supplement: Attachment — Submitted filename: Response to reviewer 25.11.docx [file pone.0316917.s003.docx]

Response to Review 25.11.2024

- Please review your reference list to ensure that it is complete and correct. If you have cited papers that have been retracted, please include the rationale for doing so in the manuscript text, or remove these references and replace them with relevant current references. Any changes to the reference list should be mentioned in the rebuttal letter that accompanies your revised manuscript. If you need to cite a retracted article, indicate the article’s retracted status in the References list and also include a citation and full reference for the retraction notice.

**We have reviewed the references, and they are complete.** **Due to the changes made to the text in response to the following comments, we have added several sources, highlighted in yellow, and updated the reference list accordingly.**

Additional Editor Comments:

(1) The abstract format for PloS One is as follows: background, methods, results, and conclusion (please add the subheadings).

**Done.**

(2) Flexibility in work hours, Consultation and support, Information about the organization, Security regarding stability, and Opportunities for volunteering could be other variables. If researchers would like to include these variables, Hypotheses could be changed (It is suggested to be deleted).

**We carefully reviewed your comment regarding the organizational variables and the suggestion to remove them. We do believe that examining organizational resources that could assist during wartime is meaningful and valuable. However, after careful consideration, we decided to narrow the focus and retained only volunteering opportunities and workplace flexibility. We revised the introduction to present the rationale for selecting these two variables and made corresponding changes throughout the study. Additionally, we updated the conceptualization of organizational variables, defining them as organizational resources rather than support.**

(3) Kindly check for sample size 373 or 374 (Table 1: total gender is 373).

**Thank you for the comment. The gender variable to include 374 participants: 261 women, 112 men, and one participant with Unreported gender.**

(4) Lines 368 (Page 70, R2), To examined the first hypothesis, the mean level of occupational burnout among 315 participants was high (M=3.95, SD=1.15) (please check grammar). Descriptive analysis could not be used to develop hypotheses. The hypothesis is for inferential statistics.

**Thank you for the comment. We replaced the hypothesis with an aim to examine and report the level of burnout in the sample during wartime**

(5) Table 2, please check for mean and SD. (five or seven-point rating scale, should not be over 5 or 7).

**Thank you for the comment. Following the guidelines for the stress and sleep disturbance questionnaires, the average total score for each respondent is presented. We have added a note below the table and highlighted this in the description of the research instruments.**

(6) Please check for hypotheses regarding Table 2 and Table 3. The hypotheses could be as follows;
H1: Stress has a positive effect on occupational burnout.
H2: Work-to-family conflict has a positive effect on occupational burnout.
H3: Family-to-work conflict has a positive effect on occupational burnout.
H4: Sleep disruption has a positive effect on occupational burnout.
H5: Routine disruption has a positive effect on occupational burnout.

**Thank you for your comment. We have carefully reviewed your suggestion and revised the hypotheses accordingly. Hypotheses 1-5 have been written as you recommended, and in the results section, we report both the correlations and the subsequent regression analysis. Additionally, we have added two new hypotheses related to organizational resources—one exploring the relationship between flexibility, volunteering, and burnout, and the other examining how these resources may moderate the relationship between work-family conflict and burnout. The results and discussion sections have been updated to reflect these changes.**

(7) Pearson (Table 2) and Regression (Table 3) differ regarding gender. It is suggested that only one analysis, Pearson or Regression, be selected (or gender should be used as the moderator). Only Table 2 could better explain the hypothesis's results.

**Thank you for your comment. In Table 1, we replaced the T-test for gender differences in burnout levels with a Pearson correlation, consistent with the regression analysis**.

(8) Table 4 is ok but only includes some predictors; it is suggested to include H1-H5.

**Following your second comment, we removed some of the variables used to measure organizational resources. We retained only with volunteering and workplace flexibility. Since flexibility was not significantly associated with burnout levels, the moderation model was conducted solely with the volunteering variable. This adjustment has been emphasized in the manuscript.**

(9) Please recheck for Tables 3 and 4 regarding H1-H5.

**Thank you for your comment. Table 3 addresses the contribution of different disruptions (resources depletion) to burnout levels, and therefore does not refer to organizational resources. Table 4 aims to examine whether the organizational resource we identified as significant for burnout can moderate the impact of work-family conflict on burnout, as emphasized in the manuscript.**

(10) The conclusion in the abstract should be summarized for H1-H5, and
the last section of the conclusion should be supported or rejected for H1-H5. Thus, please revise to H1-H5 (gender could be the moderator).

**Thank you for the comment. We made changes to the abstract accordingly**
